# Supplementary material for: Associations between Objectively Determined Physical Activity and Cardiometabolic Health in Adult Women: A Systematic Review and Meta-Analysis
Source: Biology (Basel). 2022 Jun 17;11(6):925. doi: 10.3390/biology11060925 (PMC9220764; doi:10.3390/biology11060925)
Supplement: Supplementary file 1 [file biology-11-00925-s001.zip › biology-1751013-supplementary.pdf]

Supplementary Table 1: methodological quality of studies

Table S1A Assessment of methodological quality of nonrandomized studies (Newcastle Ottawa Scale).

Study	Selection				Comparability	Outcome			Total	Quality
	Representativeness of the exposed cohort	Selection of non-exposed cohort	Ascertainment of exposure	Outcome does not present at start of study	Adjusted for age and other important covariates	Assessment of outcome	Follow-up long enough for outcomes to occur	Adequacy of follow up		
Camhi et al. 2015	-	*	*	*	**	*	n/a	n/a	6	High
Diniz et al. 2015	-	*	*	*	*	*	n/a	n/a	5	High
Graff et al. 2012	-	*	-	*	-	*	n/a	n/a	3	Low
Green et al. 2014	*	*	*	*	-	*	n/a	n/a	5	High
Hasan et al. 2018	-	*	*	*	-	*	-	*	5	High
Koniak-Griffin et al. 2014	*	*	-	*	-	*	n/a	n/a	4	High
Lecheminant et al. 2011	*	*	*	*	**	*	n/a	n/a	7	High
Loprinzi et al. 2012	*	*	-	*	**	*	n/a	n/a	6	High
Macena et al. 2021	-	*	*	*	**	*	n/a	n/a	6	High
Musto et al. 2010	-	*	-	*	-	*	-	*	4	Low
Panton et al. 2007	-	*	-	*	-	*	n/a	n/a	3	Low
Rodriguez-Hernandez et al. 2018	-	*	*	*	-	*	n/a	n/a	4	Low
Slater et al. 2021	*	*	*	*	**	*	n/a	n/a	7	High
Swartz et al. 2003	-	*	-	*	-	*	-	*	4	Low
Tabozzi et al. 2020	-	*	*	*	*	*	n/a	n/a	5	High
Vella et al. 2011	*	*	*	*	-	*	n/a	n/a	5	High
Vella et al. 2009	*	*	*	*	**	*	n/a	n/a	7	High
Zając-Gawlak et al. 2017	-	*	*	*	-	*	n/a	n/a	4	High

Notes: Representativeness of the exposed cohort: One star = truly/somewhat representative of general female adults; Selection of the non-exposed cohort: One star = drawn from the same community as the exposed cohort; Comparability: One star = study controls for age, 2nd star = study controls for other confounders; Follow-up long enough for outcomes to occur: One star = ≥12 months; Adequacy of follow up: Complete follow up/all groups had similar loss to follow-up/less than 5% of total lost to follow-up

n/a: not applicable

Table S1B Assessment of methodological quality of randomized studies (the Cochrane Collaboration's tool).

Study	Random sequence generation	Allocation concealment	Blinding of participants and personnel	Blinding of outcome assessment	Incomplete outcome data	Selective reporting	Other sources of bias	Overall Quality
Hornbuckle et al. 2012	L	L	L	L	L	L	L	High
Moreau et al. 2001	U	L	L	L	L	L	L	U
Pal et al. 2011	U	L	L	L	L	L	L	U
Sugawara et al. 2006	U	L	L	L	L	L	L	U
Sugiura et al. 2002	U	U	L	L	L	L	L	U

Note: L, low risk; H, high risk, U, unclear.

Table S1C GRADE assessment

			Quality Assessment					
	**No. of participants (#studies)**	**study design**	**Risk of bias1**	**Inconsistency2**	**Indirectness3**	**Imprecision4**	**Publication bias5**	**Absolute effect**
**Blood pressure**	113(4)	Random experiments	no serious	no serious	serious	serious	not applicable	2/4 reported increased steps/d had no effect on SBP and DBP [Hornbuckle et al., 2012; Pal et al., 2011]<br>1/4 reported increased steps/d had favorable effect on SBP, and no effect on DBP [Moreau et al., 2001]<br>1/4 reported increased PA intensity with no effect on SBP or DBP [Sugawara et al., 2006]
	147(3)	Non-random experiments	no serious	no serious	no serious	serious	not applicable	1/3 reported increased steps/d had no effects on SBP or DBP [Hasan et al., 2018]<br>1/3 reported increased steps/d had favorable effect on SBP and DBP [Swartz et al., 2003]<br>1/3 reported increased steps/d had favorable effect on SBP, but no effect on DBP [Musto et al., 2010]
	690(6)	Cross-sectional	no serious	no serious	no serious	no serious	not applicable	**Steps:** no association with SBP or DBP (3/3 Koniak-Griffin et al., 2014; Panton et al., 2007; Vella et al., 2009);<br>**TPA:** no association with SBP or DBP (1/1 Slater et al., 2021);<br>**LPA:** no association with SBP or DBP (1/1 Green et al. 2014);<br>**MVPA:** no association with SBP or DBP (3/3 Green et al., 2014; Koniak-Griffin et al., 2014; Slater et al., 2021);<br>**MVPA bouts (10min):** no association with SBP or DBP (2/2 Green et al., 2014; Koniak-Griffin et al., 2014)<br>**Meeting PA guideline (≥150 min/w MVPA):** no association with SBP or DBP (1/1 Vella et al., 2011)
**Lipid profile**	71(2)	Random experiments	no serious	no serious	no serious	serious	not applicable	1/2 reported inceased steps/d had no effects on HDL, TG, or TC [Hornbuckle et al., 2012]<br>1/2 reported increased steps/d had favorable effects on HDL and TC, had no effects on TG or LDL [Sugiura et al., 2002].

	129(2)	Non-random experiments	serious	no serious	no serious	serious	not applicable	1/2 reported increased steps/d had favorable effects on LDL [Hasan et al. 2018];<br>2/2 reported increased steps/d had no effects on TC, TG, or HDL [Hasan et al., 2018; Musto et al., 2010]
	800(8)	Cross-sectional	no serious	serious	no serious	no serious	not applicable	**Steps:**<br>*LDL:* no association [2/3 Graff et al., 2012; Koniak-Griffin et al., 2014]; unfavorable association [1/3 Panton et al., 2007];<br>*HDL:* no association [3/4 Graff et al., 2012; Koniak-Griffin et al., 2014; Panton et al., 2007]; favorable association [1/4 Vella et al., 2009];<br>*TC:* no association [2/3 Graff et al., 2012; Koniak-Griffin et al., 2014]; unfavorable association [1/3 Panton et al., 2007];<br>*TG:* favorable association [2/4 Koniak-Griffin et al., 2014; Vella et al,. 2009]; no association [2/4 Graff et al., 2012; Panton et al., 2007];<br>**TPA:** no association with LDL, HDL, TG, or TC [Slater et al. 2021];<br>**LPA:** favourable association with TG and TC; no association with HDL or LDL [Green et al., 2014];<br>**MVPA:**<br>*LDL:* no association [3/3 Slater et al., 2021; Green et al., 2014; Koniak-Griffin et al., 2014];<br>*HDL:* favorable association [2/3 Slater et al., 2021; Koniak-Griffin et al., 2014); no association [Green et al., 2014];<br>*TC:* no association [2/3 Slater et al., 2021; Green et al., 2014]; unfavorable association [1/3 Koniak-Griffin et al., 2014];<br>*TG:* no association [3/3 Slater et al., 2021; Green et al., 2014; Koniak-Griffin et al., 2014]<br>**MVPA bouts (10min):** no association with LDL, HDL, TG, or TC [2/2 Green et al., 2014; Koniak-Griffin et al., 2014]<br>**Meeting PA guideline (≥150 min/w MVPA):** favorable association with TG and TC; no association with LDL or HDL [Vella et al. 2011].

**Carbohydrate metabolism**	68(2)	Random experiments	serious	no serious	no serious	serious	not applicable	1/2 reported increased steps/d had no effects on HbA1c [Hornbuckle et al., 2012];<br>1/2 reported increased steps/d had no effects on FPG or HOMA-IR [Moreau et al., 2001];
	157(4)	Non-random experiments	serious	no serious	no serious	serious	not applicable	**Increased steps:**<br>FPG: favorable association [1/3 Musto et al. 2010]; no association [2/3 Hasan et al., 2018; Swartz et al., 2003]<br>PPG: favorable association with 2h-PPG [1/1 Swartz et al. 2003];<br>HOMA-IR: no association [Hasan et al., 2018]<br>2h-AUCglc: favorable association [1/1 Swartz et al. 2003]<br>**Increased %MVPA:** favorable association with 4h-PPG, 2h-AUCglc; no association with peak PGCL [1/1 Rodriguez-Hernandez et al., 2018];<br>**Increased %LPA:** no association with PPG, or 2h-AUCgle [1/1 Rodriguez-Hernandez et al., 2018].
	1135(11)	Cross-sectional	no serious	no serious	no serious	no serious	no serious	**Steps:**<br>*FPG:* no association [4/4 Graff et al., 2012; Koniak-Griffin et al., 2014; Tabozzi et al., 2020; Vella et al., 2009];<br>*PPG:* no association [2/2 Graff et al., 2012; Tabozzi et al., 2020];<br>*HbA1c:* no association [1/1 Panton et al., 2007]<br>*HOMA-IR:* favorable association [1/2 Graff et al., 2012]; no association [1/2 Macena et al., 2021]<br>**TPA: no association with FPG, HbA1c, or HOMA-IR [1/1 Slater et al., 2021]**<br>**LPA:**<br>*HOMA-IR:* no association [2/2 Macena et al. 2021; Green et al. 2014];<br>*FPG:* no association [1/1 Green et al. 2014]<br>**%LPA:** no association with FPG or PPG [1/1 Tabozzi et al., 2020]<br>**MPA:** no association with HOMA-IR [2/2 Lecheminant et al., 2011; Macena et al., 2021];

								**%MPA and %VPA:** no association with FPG, favorable association with PPG [1/1 Tabozzi et al., 2020];<br>**MVPA:**<br>*FPG:* no association [4/4 Green et al., 2014; Koniak-Griffin et al., 2014; Slater et al., 2021; Tabozzi et al., 2020]<br>*PPG:* favorable association with peak PPG [1/1 Tabozzi et al., 2020]<br>*HbA1c:* no association [1/1 Slater et al., 2021]<br>**MVPA bouts (10min):** no association with FPG [2/2 Green et al., 2014; Koniak-Griffin et al., 2014]; favorable association with HOMA-IR [1/1 Green et al., 2014]<br>**Meeting PA guideline (≥150 min/w MVPA):**<br>*FPG:* no association [1/1 Vella et al., 2011];<br>*HOMA-IR: no association [3*/3 Lecheminant et al., 2011; Diniz et al., 2015; Vella et al., 2011]
**Endocrine regulators**	24(1)	Random experiments	serious	no serious	no serious	serious	not applicable	Increased steps had no effect on fasting insulin [Moreau et al., 2001]
	70(2)	Non-random experiments	serious	no serious	no serious	serious	not applicable	2/2 reported increased steps had no effect fasting insulin or postprandial insulin [Hasan et al., 2018; Swartz et al., 2003]
	502(5)	Cross-sectional	no serious	serious	no serious	no serious	not applicable	**Steps/d:** favorable association with fasting insulin and postprandial insulin [1/1 Graff et al., 2012];<br>**TPA:** favorable association with fasting insulin [1/1 Slater et al., 2021];<br>**LPA:** no association with fasting insulin [1/1 Green et al., 2014];<br>**MVPA:** no association with fasting insulin [2/2 Green et al., 2014; Slater et al., 2021]<br>**MVPA bouts (10min):** favorable association with fasting insulin [1/1 Green et al., 2014]<br>**Meeting PA guideline (≥150 min/w MVPA):** no association with fasting insulin [2/2 Diniz et al., 2015; Vella et al., 2011]

**Inflammation marker**	44(1)	Random experiments	no serious	no serious	no serious	serious	not applicable	1/1 reported increased steps/d had no effect on CRP [Hornbuckle et al., 2012]
	409(4)	Cross-sectional	no serious	serious	no serious	no serious	not applicable	**Steps:** no association with CRP [1/1 Panton et al., 2007]<br>**TPA:** favorable with CRP [1/1 Slater et al., 2021]<br>**LPA:** no association with TNF-alpha, CRP, or IL-6 [1/1 Green et al., 2014]<br>**MVPA:** favorable association with TNF-alpha and CRP, no association with IL-6 (1/1 Green et al. 2014); unfavorable with CRP [1/1 Slater et al., 2021]<br>**MVPA bouts (10min):** favorable association with CRP, no association with IL-6 or TNF-alpha [1/1 Green et al., 2014]<br>**Meeting PA guideline (≥150 min/w MVPA):** no association with TNF-alpha [1/1 Diniz et al., 2015)
**MS**	52(1)	Non-random experiments	no serious	no serious	no serious	serious	not applicable	1/1 reported increased steps/d had favourable effect on MS score (Hasan et al. 2018)
	708(4)	Cross-sectional	no serious	no serious	no serious	no serious	not applicable	**Steps:** favorable association [1/2 Zając-Gawlak et al., 2017]; no association [1/2 Camhi et al., 2015];<br>**LPA:** favorable association [Camhi et al., 2015];<br>**MPA, VPA, MVPA bouts (10min) and TPA:** no association [Camhi et al., 2015];<br>**MVPA:** favorable association [1/1 Loprinzi et al., 2012]; no association [Camhi et al., 2015].

Note:

1. We downgraded one level if 50% to 75% of studies were low quality and two levels if more than 75% of studies were low quality.
2. We downgraded one level for inconsistency of findings were highly inconsistence
3. We downgraded one level for indirectness if studies examined various types of PA intervention and provided indirect evidence or made indirect comparisons.
4. We downgraded one level for imprecision if the number of participants was less than 400.
5. Publication bias test was not applicable when n<10.

CRP, C-reactive protein; DBP, diastolic blood pressure; FPG, fasting glucose; HbA1c, glycosylated hemoglobin; HDL, high density lipoprotein; HOMA-IR, homeostasis model assessment of insulin resistance; IL-6, interleukin-6; LDL, low density lipoprotein; LPA, low intensity physical activity; MPA, moderate intensity physical activity; MS, metabolic syndrome; MVPA, moderate to vigorous intensity physical activity; PPG, postprandial glucose; SBP, systolic blood pressure; TC, total cholesterol; TG, triglyceride; TNF-alpha, tumor necrosis factor-a; TPA, total physical activity; VPA, vigorous physical activity.
